# Multiple Myeloma Patients Undergoing Carfilzomib: Development and Validation of a Risk Score for Cardiovascular Adverse Events Prediction

**DOI:** 10.3390/cancers13071631

**Published:** 2021-04-01

**Authors:** Anna Astarita, Giulia Mingrone, Lorenzo Airale, Fabrizio Vallelonga, Michele Covella, Cinzia Catarinella, Marco Cesareo, Giulia Bruno, Dario Leone, Carlo Giordana, Giusy Cetani, Marco Salvini, Francesca Gay, Sara Bringhen, Franco Rabbia, Franco Veglio, Alberto Milan

**Affiliations:** 1 Hypertension Unit, Department of Medical Sciences, Division of Internal Medicine, AO “Città della Salute e della Scienza” University Hospital, 10126 Turin, Italy; giulia.mingrone@edu.unito.it (G.M.); lorenzo.airale@unito.it (L.A.); fabrizio.vallelonga@unito.it (F.V.); mcovella@ausl.vda.it (M.C.); cinzia.catarinella@edu.unito.it (C.C.); marcoriccardo.cesareo@unito.it (M.C.); giulia.bruno87@gmail.com (G.B.); dgleone@live.it (D.L.); carlo.giordana@edu.unito.it (C.G.); franco.rabbia@libero.it (F.R.); franco.veglio@unito.it (F.V.); alberto.milan@gmail.com (A.M.); 2 Myeloma Unit, Department of Medical Sciences, Division of Hematology, AO “Città della Salute e della Scienza” University Hospital, 10126 Turin, Italy; g.cetani@gmail.com (G.C.); marcosalvini83@gmail.com (M.S.); francesca.gay@unito.it (F.G.); sarabringhen@yahoo.com (S.B.)

**Keywords:** multiple myeloma, carfilzomib, cardiovascular adverse events, cardiovascular risk assessment, arterial hypertension, echocardiography, global longitudinal strain, pulse wave velocity

## Abstract

**Simple Summary:**

Despite the relationship between Carfilzomib (CFZ) therapy in multiple myeloma (MM) and cardiovascular adverse events (CVAEs), no specific validated protocols on cardiovascular risk assessment are available. In this prospective study, we investigated major predictors of CVAEs prior to starting CFZ, applying the European Myeloma Network management protocol (EMN). Five predictors were identified: office systolic blood pressure, 24-h blood pressure variability, left ventricular hypertrophy, pulse wave velocity value and global longitudinal strain. The resulting ‘CVAEs risk score’ defined a low- and a high-risk group (negative predicting value for the high-risk group of 90%). 62 patients experienced one or more CVAEs: 17 major and 45 hypertension-related events. In conclusion, CVAEs are frequent and a specific management protocol is required. The EMN protocol and ‘CFZ risk score’ proved to be effective in estimating the baseline risk of CVAEs during CFZ therapy in MM patients, targeting the appropriate follow-up.

**Abstract:**

Cardiovascular adverse events (CVAEs) are linked to Carfilzomib (CFZ) therapy in multiple myeloma (MM); however, no validated protocols on cardiovascular risk assessment are available. In this prospective study, the effectiveness of the European Myeloma Network protocol (EMN) in cardiovascular risk assessment was investigated, identifying major predictors of CVAEs. From January 2015 to March 2020, 116 MM patients who had indication for CFZ therapy underwent a baseline evaluation (including blood pressure measurements, echocardiography and arterial stiffness estimation) and were prospectively followed. The median age was 64.53 ± 8.42 years old, 56% male. Five baseline independent predictors of CVAEs were identified: office systolic blood pressure, 24-h blood pressure variability, left ventricular hypertrophy, pulse wave velocity value and global longitudinal strain. The resulting ‘CVAEs risk score’ distinguished a low- and a high-risk group, obtaining a negative predicting value for the high-risk group of 90%. 52 patients (44.9%) experienced one or more CVAEs: 17 (14.7%) had major and 45 (38.7%) had hypertension-related events. In conclusion, CVAEs are frequent and a specific management protocol is crucial. The EMN protocol and the risk score proved to be useful to estimate the baseline risk for CVAEs during CFZ therapy, allowing the identification of higher-risk patients.

## 1. Introduction

Carfilzomib (CFZ) is an irreversible second-generation proteasome inhibitor which has shown a significant improvement in survival rates in relapsed and/or refractory multiple myeloma (MM) [1]. Since its approval, several reports [2,3,4] highlighted a connection between use of CFZ and cardiovascular adverse events (CVAEs), due to an overlapping of risk factors, involving irreversible proteasome inhibitor cardiotoxicity and the wide-range of MM-related comorbidities. Heart failure, arrhythmias, acute coronary syndromes, sudden cardiac death and hypertensive events have all been described [5,6]. A review and meta-analysis, including phase 1 to 3 prospective trials on CFZ therapy in MM patients, estimated an 18.1% incidence of CVAEs during CFZ, with a high frequency of hypertensive events and heart failure [7]. 

In spite of the increased potential for life-threatening CVAEs, no validated management protocols on cardiovascular risk assessment are available, making it challenging to assess the individual cardiovascular risk profile and to estimate the probability of CVAEs during CFZ therapy. Furthermore, the risk factors and real incidence of each type of CVAE is unknown, due to lack of ‘real-life’ studies. The key objective of this prospective study was to evaluate the effectiveness of the multimodality assessment protocol proposed by the Consensus Paper of the European Myeloma Network (EMN) [8] for the cardiovascular risk assessment of MM patients undergoing CFZ therapy. Major predictors of CVAEs were identified and a risk score prediction model for CVAEs was developed in order to identify patients at higher risk for cardiovascular events, and consequently with higher risk for discontinuation of CFZ therapy, who require a closer and tailored follow-up. The type and incidence of CVAEs were investigated.

## 2. Materials and Methods

This cohort prospective study was conducted at the third level Hypertension Unit and Centre for Cardiovascular diseases of ‘Città della Salute e della Scienza’ Hospital in Turin, Italy. Patients with MM who had indication for CFZ were consecutively enrolled between January 2015 and March 2020. Patients previously treated with CFZ, those affected by light chain cardiac amyloidosis (assessed by end-organ biopsy or cardiac magnetic resonance imaging) and those refusing informed consent were excluded. 

### 2.1. Baseline Assessment before Starting CFZ

In accordance with the European Myeloma Network Protocol [8], before starting CFZ therapy, patients underwent a detailed baseline assessment, including cardiovascular history, physical examination, office blood pressure measurements, ambulatory blood pressure monitoring (ABPM), 12-leads ECG, trans-thoracic echocardiography with global longitudinal strain assessment (GLS) and estimation of arterial stiffness through pulse wave velocity (PWV). Office blood pressure (BP) was recorded by an automatic sphygmomanometer: three consecutives BP measurements 1–2 min apart (supine, standing, sitting) were performed and the mean value was used (Omron, M10-IT model; Omron Healthcare Co.; Kyoto, Japan) [9]. ABPM was performed with a 24-h recording (reading taken every 15 minutes throughout the day and every 30 minutes during the night), using a validated measuring device (Takeda TM2430; A&D Company Ltd, Tokyo, Japan). The following measurements were extracted: systolic BP, diastolic BP, mean BP and standard deviation during the 24-hours, during the day and night, blood pressure variability (BPV) (derived from the average of night-time SD corrected for the respective duration of night), pulse pressure (difference between systolic BP–diastolic BP) and dipping value (derived using the formula (1-(SBP night/SBP day)) [9]. Trans-thoracic echocardiography was performed placing the patient in left lateral decubitus position (iE33 ultrasound machine, Philips Medical System, Andover, MA, USA). In parasternal long-axis view, left ventricular (LV) diameters and wall thickness were measured (deriving LV geometry through the Deveraux formula indexed to both body surface area and height elevated to 2.7). LV hypertrophy (LVH) was defined in presence of LV mass ≥115 g/m2 (≥49 g/m2.7) and ≥95 g/m2 (≥47 g/m2.7) in men and women, respectively. LV ejection fraction (LVEF) and LV volumes were assessed from the apical 4- and 2-chamber views indexed to body surface area (Simpson’s Biplane technique) [10]. The LV diastolic function was assessed using the combination of TDI (e’ septal and lateral anulus mitral waves), Pulsed Doppler (E and A mitral valve waves), tricuspid regurgitation peak velocity, left atrial volume (indexed to body surface area) and E/e’ ratio (according to the current recommendations) [11]. The global longitudinal strain (GLS) was performed using the speckle tracking analysis system, using dedicated software (Automated Cardiac Motion Quantification, QLAB Cardiac Analysis, Philips, Andover, MA, USA). The GLS was computed offline from standard 2D images of the LV apical views (4-,3- 2- chamber), tracing the endocardium border by semi-automatic system with manual adjustment when needed, performed by standardized protocols [12]. Arterial stiffness estimation was performed through the carotid-femoral pulse wave velocity (PWV) using a validated device (Sphygmocor system Atcor Medical, Sydney, Australia). A single applanation tonometer was used to obtain and record carotid and femoral pulse waveforms, performed by expert operators. The PWV value was derived from the formula: distance covered by the pulse wave divided by the delay time (measured between the feet of the two waveforms). The mean of at least two PWV measurements (considering a standard deviation for each record < 10%), were considered valid for the analysis, according to the current guidelines [13]. During the first visit, patients with either office or out-of-office blood pressure values in the high/normal range and those with arterial hypertension, according to the ESH recommendations [9], were advised to start anti-hypertensive treatment or to optimize the previous anti-hypertensive therapy in order to obtain blood pressure control before starting CFZ therapy. CFZ-based regimen, timing and dosing were decided by the hematologists.

### 2.2. Follow-Up Assessments and CVAEs

Follow-up assessments were performed every six months and/or at the time of any suspected CVAE. The type and the incidence of CVAEs were checked during the clinical evaluations, through periodic review of patients’ electronic reports and by phone interviews. Patients were followed until the end of CFZ therapy. CVAEs were ranked into two subgroups: major CVAEs and arterial hypertension-related CVAEs. Specifically, we considered as ‘major CVAE’ all the events having a cardiovascular origin, excluding analysis which could not be expressively related to cardiovascular causes (CVAEs’ definitions used are available in the Appendix A). CVAE events were graded according to the Common Terminology Criteria for Adverse Events version 5.0 (CTCAE 5.0) [14].

### 2.3. Statistical Analysis

Difference in baseline parameters between patients who experienced CVAEs and patients who did not experience CVAEs was investigated by the chi-square test/Fisher’s exact test for categorical variables and by the unpaired *t*-test/Mann Whitney test for continuous variables, as appropriate. A two-sided *p* value less than 0.05 was used as the level of statistical significance. The univariate binary logistic regressions were used to investigate the hazard ratio of each variable for CVAEs. The analysis was performed using a dedicated software (IBM SPSS Statistics, Version 22.0.0.0, IBM Corp., Armonk, NY, USA).

### 2.4. CFZ CVAEs Risk Score Analysis

The CFZ CVAEs Risk Score was developed according to the CHARMS Recommendations [15] and adheres to the TRIPOD Statement for Prediction Model [16]. The analysis was performed using a dedicated software (R: A Language and Environment for Statistical Computing, v.4.0.0 for Mac OSX, R Core Team, Vienna, Austria). Missing data (3.8% of the 1276 total variables) were estimated by multiple imputations system as recommended [15,17]. Regression analysis was performed by two penalized models: the first, with the aim of reducing the set of covariates [18], and the second with the aim of building a risk predictor system, the ‘CFZ CVAEs risk score’. The score was internally validated by bootstrapping according to the CHARMS Recommendations [15]: 1000 random samplings with replacement from the original sample were performed in order to assess the discrimination and the calibration of the score. Discrimination was calculated with C statistic, and calibration was assessed using the calibration plot and the calibration slope. The optimal threshold was identified by imposing a sensibility value ≥ 90%.

## 3. Results

### 3.1. Baseline Characteristics and Cardiovascular Risk Factors

**Figure 1:**
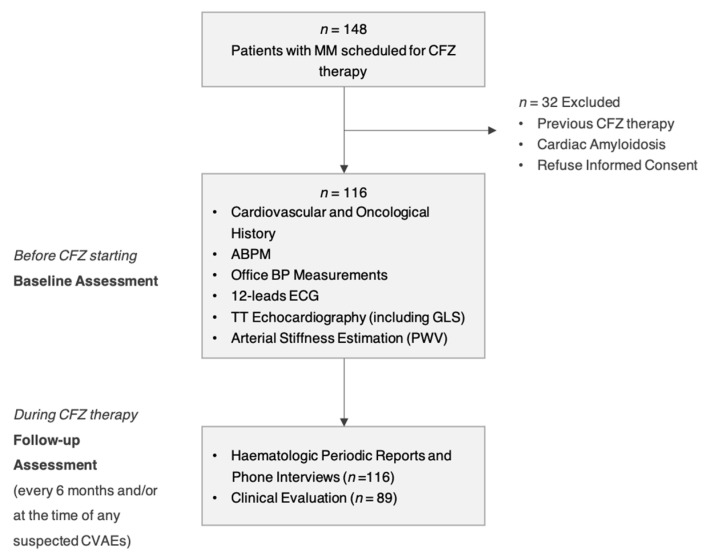
Flow-chart of the Study Protocol.

Of 148 patients, 116 matched the inclusion criteria and they were enrolled (Figure 1 shows the flow-chart of the protocol). The baseline characteristics of the study sample are listed in Table 1. Tobacco use (50%) and known arterial hypertension (41.1%) were the most common cardiovascular (CV) risk factors. According to office blood pressure values and/or ABPM, 51.7% met the criteria for arterial hypertension, requiring the introduction of a new anti-hypertensive treatment or a modification of their previous therapy. The prevalence of asymptomatic hypertension-mediated organ damage (HMOD) was substantial: 20.7% had left ventricular hypertrophy at echocardiography evaluation, 20.7% had GLS impairment (defined as GLS ≤ 20%) and 26.7% showed increased arterial stiffness (PWV ≥ 9 m/s). The ABPM was available for 107 patients (nine patients had inappropriate recording/invalid result < 70% day/night measurements), the GLS assessment for 115 patients (one patient had echo images with poor quality) and PWV assessment for 103 patients (13 patients had less than two valid measurements due to tachycardia, extrasystoles, or technical difficulty due to overweight).

### 3.2. CFZ Therapy Regimen: Timing and Dose

The median duration of CFZ therapy was 8.6 months (0.1 to 52.8) for a median cumulative dose of 2781.9 (29.4–1323.5) mg of CFZ (computed at the end of the planned therapy or at the end of the study if CFZ was still ongoing). The most common regimen was a combined protocol with lenalidomide and dexamethasone (53 patients; 45.6%) [19]. 79 (86.1%) patients received up to 36 mg/m2 of CFZ dose per administration, 32 (27.5%) up to 56 mg/m2 and three (2.6%) up to 70 mg/m2, without differences observed in CVAEs between groups (*p* > 0.05 for all comparisons). At the end of the study, 89 patients (76.2%) had discontinued CFZ therapy. Causes for discontinuation included disease progression (*n* = 64), patients’ decision (*n* = 6), non-cardiovascular toxicity (renal, hematological, gastrointestinal) (*n* = 12) and (*n* = 7) cardiovascular toxicity (*n* = 6 for major CVAEs and *n* = 1 for uncontrolled hypertension); moreover two patients required a dose reduction because of cardiovascular toxicity (Table S1 details the CFZ regimens of patients enrolled, Table S2 details the cumulative dosages of CFZ at first CVAE, available in Supplementary Materials).

### 3.3. Rates of CVAEs: All-Types, Major and Hypertension-Related CVAEs

The subtypes of CVAE occurring during CFZ therapy are listed in Table 2. During a median follow-up of 10.8 months, 52 patients (44.9%) experienced one or more CVAEs, with 30.9% grade 3 or greater in severity (CTCAE ≥ 3). 17 subjects (14.7%) experienced major CVAEs, with four patients (23.5%) experiencing more than one major CVAE. Arrhythmias (41.2%), acute coronary syndromes (23.5%) and post-infusion dyspnea (23.5%), were the most commonly represented; one sudden cardiac death occurred after 22 months of CFZ therapy. 45 patients (38.7%) experienced hypertension-related CVAEs, of which 72% were grade 1-2 in severity. 10 patients experienced both major and hypertensive CVAEs, with 13 CVAEs grade ≥ 3 in severity. 27 patients (51.9%) experienced CVAEs within the first three months of treatment. 

### 3.4. Comparison between No-CVAEs and CVAEs Group: Baseline CV Parameters

At baseline, patients experiencing CVAEs had higher BP values and higher indices of BP variability at office and at ABPM, specifically higher systolic and diastolic office blood pressure values, higher daytime systolic BP and BP variability at ABPM. At echocardiographic evaluation, the CVAEs group had a significantly higher left ventricular mass and a reduction in GLS mean value, with no difference in LVEF. Furthermore, patients who experienced CVAEs had increased arterial stiffness at baseline, as demonstrated by the higher PWV value. No meaningful differences in age, sex, anthropometric variables, previous cardiovascular history, ECG, MM duration and previous oncological treatments (Anthracyclines, Alkylating agents, Immunomodulating agents, Bortezomib, All) were observed between the two groups. The baseline values that best predicted the incidence of CVAEs during CFZ therapy (variables made categorical using the Youden index and included in a logistic regression) were: office SBP ≥ 131.5 mmHg (OR = 5.41; *p* = 0.001), BP variability ≥ 10 at ABPM (OR = 5.071; *p* = 0.002), presence of LVH at echocardiography (OR = 3.056; *p* = 0.021), PWV ≥ 8.75 m/s (OR = 5.13; *p* = 0.000). Table 3 details the comparison of the main baseline parameters between no-CVAEs and CVAEs group, and Table S3 details the remaining ones (available in Supplementary Materials).

### 3.5. CFZ CVAEs Risk Score

Firstly, we performed a penalized regression including variables which showed a *p* value < 0.005 at the univariate logistic regression together with those for which a predictive value was to be expected based on current literature (age, sex, diabetes, ischemic heart disease, chronic kidney disease, office SBP, BPV, PWV, LVEF, LVH and GLS). According to the CHARMS Recommendations [15], to comply with an event per variable (EPV- number of events/number of variables) > 10, we performed a variable selection by penalized logistic regression (elastic net regression) fixing a maximum of five output variables with non-zero coefficient. SBP office, BPV, PWV, LVH and GLS had their coefficients not forced to zero, therefore they were included in the second penalized regression. Coefficients extracted from this model were used to calculate the CFZ CVAEs Risk Score equation:

CFZ CVAEs Risk Score = −2.84666907 + (0.08244733∙BPV) + (0.17791129∙PWV) + (0.57680056∙LVH) + (−0.06201887∙GLS) + (0.01282460∙office SBP)

Therefore, the predicted risk of event was:

$$Score=\frac{e^{Score\;Risk}}{1+e^{Score\;Risk}}$$

**Figure 2:**
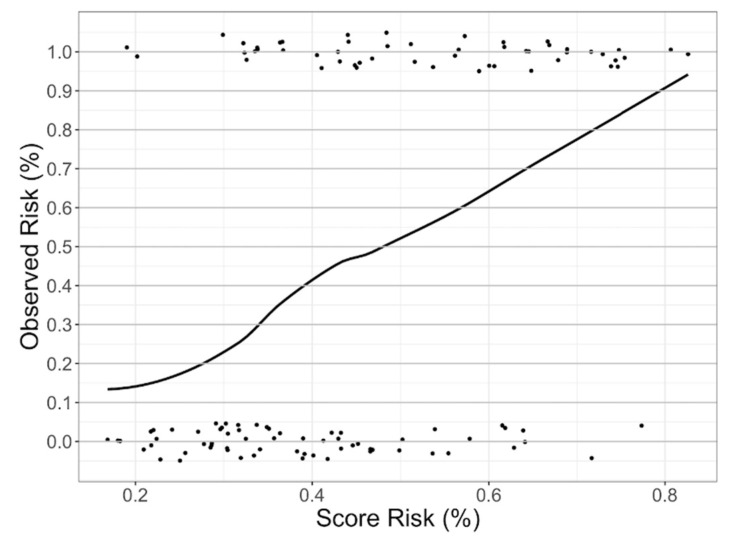
Probability of incurring CVAEs related to increasing of carfilzomib (CFZ) CVAEs Risk Score. Vertical axis = observed CVAE events (%); Horizontal axis = predicted CFZ CVAE risk (%). The upper points represent patients who experienced CVAEs; the lower points represent patients who did not experienced CVAEs.

**Figure 3:**
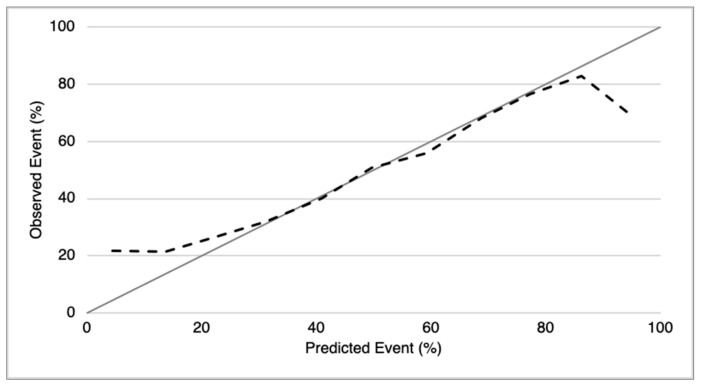
Smooth Calibration plot for the validation of CFZ CVAEs Risk Score. Comparison between observed and predicted CVAEs based on bootstrap. The grey line represents the perfect condition in which the observed CVAEs are equal to predicted CVAEs; the black line represents the CVAEs observed in our population.

**Figure 4:**
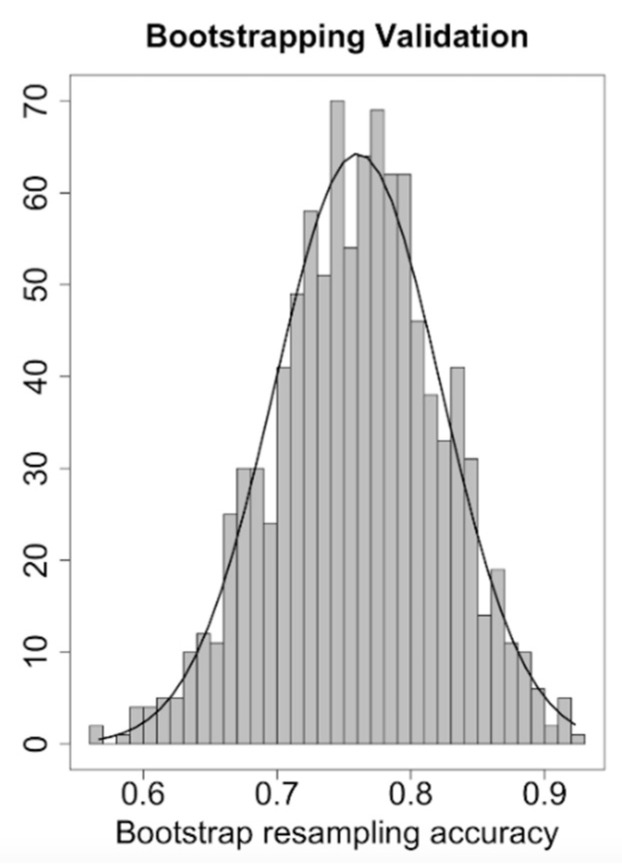
CFZ CVAEs Risk Score Discrimination plot.

**Figure 5:**
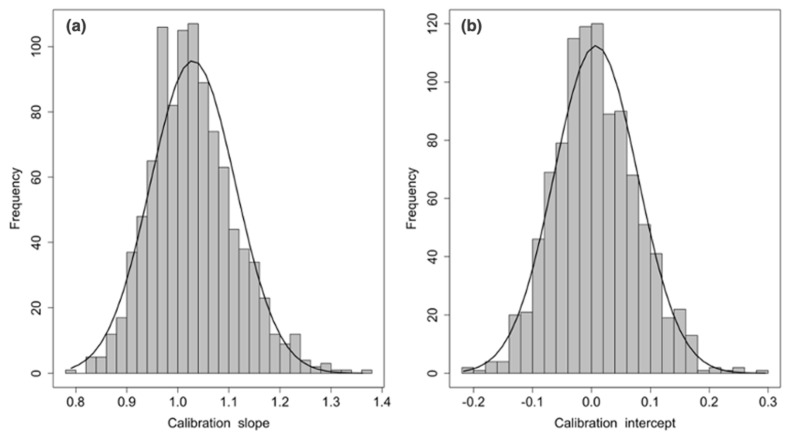
CFZ CVAEs Risk Score Calibration: (**a**) calibration slope and (**b**) calibration intercepts.

We pre-determined the sensibility value to be ≥90% and obtained a risk-score cut-off of 32% to divide the study sample into a low- and a high-risk group for CVAEs (respectively 30 and 86 patients). The resulting negative predictive value for CVAEs in the high-risk group was 90% (with a specificity of 42% and AUC of 0.76). Figure 2 shows the probability of incurring CVAEs to increasing of CFZ CVAEs Risk. 3 patients in the low-risk group and 49 patients in the high-risk group experienced CVAEs. The validation, in absence of another different sample, was conducted through the method the internal validation, using the bootstrap methodology (Figure 3 and Figure 4). Mean C statistic was 0.76 ± 0.06: the smooth calibration curve fit the perfect condition (observed = expected), with a calibration slope close to 1 (1.0 ± 0.1) and a calibration intercept close to 0 (0.0 ± 0.1), as shown in Figure 5.

## 4. Discussion

Carfilzomib therapy is closely related to high risk of CVAEs in MM patients, with a large variability in type and severity of events. It is clear that a cardiovascular baseline assessment is pivotal in MM patients undergoing drugs with a potentially cardio-toxic profile, such as CFZ. In this regard, previous cardiovascular risk assessment protocols have been proposed in cancer patients undergoing cardio-toxic treatments [20,21,22]; however, no prospective studies have validated their effectiveness in MM. Besides this, the same ESH Systematic Coronary Risk Evaluation (SCORE) [23] might be inadequate to estimate the global CV risk in this complex category of patients. In this study, MM patients scheduled for CFZ therapy underwent a multisystemic assessment according to the Consensus Paper of the European Hematology Association joint with the European Myeloma Network [8]. By applying this protocol, at baseline, MM patients showed a high CV risk profile, as demonstrated by the large prevalence of uncontrolled blood pressure at office and/or ABPM and by the high proportion of asymptomatic target organ damage. However, currently there is limited evidence regarding predictors of CVAEs with conflicting results, due to the lack of prospective studies [22]. Among baseline parameters, we investigated which variables before starting CFZ could predict the risk of CVAEs occurring. Through consecutive penalized regressions, we identified five independent predictors of CVAEs which were included in the model ‘the CVAEs risk score’: (1) office SBP value, (2) BPV value at ABPM, (3) GLS value, (4) presence/absence of LVH at echocardiography and (5) PWV value at arterial stiffness estimation. In the score, two variables referred to blood pressure control: SBP at office measurements and BPV at out-of-office recording. The systematic assessment of ABPM confirmed in MM patients the association between a high BPV, cardiovascular events and disease mortality [24,25], previously described in hypertensive patients. Regarding the GLS, we found that its reduction at baseline was a leading risk factor for CVAEs, as opposed to LVEF, suggesting the relevant prognostic value of functional LV baseline evaluation, even if the GLS assessment is not systematically recommended [22]. Hence, the GLS confirmed its superiority in detection of early subclinical LV myocardial injury, as previously demonstrated in the general population [26,27]. Concerning the PWV, in our study the increase of arterial stiffness, was an independent predictor of CVAEs [28]. Although it is a known CV risk factor, recommended as a part of hypertensive patients, until now its role in cancer patients was not well established. These five parameters were included in the model in which the CV risk depends on a complex interaction between the predictors’ value that relate to one another. Using the CVAEs risk score, patients were distinguished into a low and a high-risk group of incurring a cardiovascular event (major and/or hypertensive). During the first visit, the application of this score by the clinician, might be a useful tool to identify patients at high-risk of incurring a CVAE, and consequently discontinuing CFZ therapy, who might benefit from specific risk mitigation strategies and follow-up. Regarding the comparative analysis between no-CVAEs and CVAEs group, we described differences in daytime SBP, mean 24 h SD, left ventricular mass and others; however, the overlap between ranges of many of these does not allow their use in clinical practice at this moment. 

Regarding the analysis of CVAEs, we fully described major and hypertension-related CVAEs, proving that up to 45% of patients undergoing CFZ therapy experienced CVAEs. This data is consistent with the PROTECT study [5] which showed an incidence of 50.7% cardiovascular events, but in contrast with other clinical trials, in which the rate of CVAEs ranged from 18% to 22% [7,29,30,31]. These results might be explained by the real-life design of our study and of Cornell’s report, including patients with comorbidities of varying severity, and by the specific focus on hypertensive CVAEs. Indeed, we observed a greater incidence of hypertensive CVAEs than previously reported: up to 39% of patients experienced one or more hypertensive events. Conversely to Cornell at al., this result was due to the choice of also including 1–2 degree hypertension events in order to be more sensible in identifying patients with a potential risk of developing aHMOD and future CV events. However, despite the high rate of CVAEs observed, only few patients required a discontinuation of CFZ therapy or a reduction of drug dosage. In fact, the majority of CVAE events were transient with low severity; in particular, among hypertensive events, only one patient discontinued CFZ for uncontrolled blood pressure. For all the remaining cases, patients continued CFZ therapy after an intensification of the hypertensive treatment. Despite the accurate recognition and the early-stage treatment of patients with arterial hypertension, a high rate of hypertensive events was observed, suggesting that the optimization of blood pressure values might be not sufficient. Periodic monitoring of BP values and frequent rechecking of adequate blood pressure control seems to be essential during CFZ therapy. In summary, based on the results, the application in clinical practice of the standardized protocol of the EMN to MM patients scheduled for CFZ therapy allows the correct estimation of the CV risk profile. The evaluation of the blood pressure (office and out of office), echocardiography (including LVH and GLS) and arterial stiffness (through the PWV) are useful to calculate the ‘CVAEs risk score’ in order to identify patients at higher risk of CVAEs during CFZ therapy, and needing a tailored follow-up.

Our study has several limitations. Firstly, the relatively small sample of the cohort without a control arm did not allow the detection of potential confounding factors. Due to the lack of sufficient data on laboratory biomarkers (NT-proBNP and cardiac troponins), it was not possible to test their prognostic significance. Regarding the tests done during the comprehensive baseline evaluation, the ABPM, the GLS and PWV assessment were not available for all patients due to technical reasons. Furthermore, the evaluation of arterial stiffness through the PWV assessment is not available in all Centers and this could limit the clinical applicability of the score. Concerning the consistent incidence of hypertensive CVAEs observed, this may have been affected both by the optimization of anti-hypertensive treatment prior to CFZ therapy and by the accurate detection of all hypertensive events during treatment, due to the specialization of our setting (Hypertension Unit). Another limitation was the absence of standardized dosing of the CFZ regimen that might explain the absence of relationship between dosing and CVAEs. Concerning the risk score, we underline two limitations: the model had to be internally validated due to the lack of an independent data sample and, in order to prevent reduction in the patient cohort, we computed missing data with a multiple imputation system. Due to these limitations, further studies are needed to validate the model on an independent larger sample.

## 5. Conclusions

CVAEs are frequent during CFZ therapy in MM and a specific management protocol is crucial. The application of the standardized protocol proposed by the European Myeloma Network and the use of the ‘CFZ CVAEs risk score’ proved to be effective in estimating the baseline risk for CVAEs during CFZ therapy, allowing for a patient-tailored follow-up and for the adoption of effective risk mitigation strategies.

## Figures and Tables

**Table 1:** Baseline population characteristics.

Characteristics	Overall Population No = 116
*General*	
Age, median (SD), years	64.53 ± 8.42
Male sex, No. (%)	65 (56)
*Individual CV risk factors, No (%)*	
Tobacco use (prior/current)	58 (50)
Obesity (BMI ≥ 30)	34 (29.3)
Known Arterial Hypertension	48 (41.4)
Diabetes	12 (10.3)
Chronic renal failure (eGFR < 60 mL/m)	22 (19)
Ischemic heart disease	4 (3.4)
Previous episodes of atrial fibrillation	2 (1.7)
Dyslipidaemia	16 (13.8)
Previous stroke	0 (0)
*Familiar CV risk factors, No (%)*	
Ischemic heart disease	26 (22.4)
Stroke	13 (11.2)
Diabetes	6 (5.2)
Arterial Hypertension	15 (12.9)
*Anti-hypertensive drugs, No (%)*	
Beta-blockers	26 (22.4)
ACE-inhibitors/angiotensin receptor blockers	59 (50.9)
Thiazide diuretics/Loop diuretics	25 (21.6)
Aldosterone receptor antagonists	2 (1.7)
Calcium channel blockers	25 (21.6)
*Office BP values*	
SBP, mean (SD), mmHg	129.4 ± 17.8
DBP, mean (SD), mmHg	76.9 ± 11.2
*ABPM* *	
Daytime SBP, mean (SD), mmHg	125.0 ± 13.2
Daytime DBP, mean (SD), mmHg	75.1 ± 8.9
24 h SBP, mean (SD), mmHg	120.9 ± 12.7
24 h DBP, mean (SD), mmHg	71.7 ± 8.1
24 h MBP, mean (SD), mmHg	88.5 ± 9.1
*HMOD, No (%)*	
LVH	24 (20.7)
GLS value ^†^ ≤ 20 %	24 (20.7)
PWV value ^‡^ ≥ 9 m/s	31 (26.7)
*Oncological History*	
Median MM disease duration, months	51.1
Carfilzomib line therapy, number	3 ± 1.6
Previous therapies ^ˆ^, No. (%)	
Anthracyclines	31 (26.7)
Alkylating agents	91 (78.4)
Immunomodulating agents	86 (74.1)
Bortezomib	97 (83.6)

Mean values estimated in: * 107 patients; ^†^ 115 patients; ^‡^ 103 patients. ^ˆ^ patients were mostly treated with multiple therapies, hence total % might amount to > 100. BMI = body mass index; eGFR = estimated glomerular filtration rate; ABPM = ambulatory blood pressure monitoring; SBP = systolic blood pressure; DBP = diastolic blood pressure; MBP = mean blood pressure; LVH = left ventricular hypertrophy (male LV mass ≥ 105 g/m^2^; female LV mass ≥ 95 g/m^2^); GLS = global longitudinal strain; PWV = pulse wave velocity; HMOD = hypertension mediated organ damage; MM = multiple myeloma.

**Table 2:** Lists of all-types of CVAE, major CVAEs and hypertension-related CVAEs.

CVAE	Patients *n* = 116 No. (%) *	No. CVAEs	
		Grade 1–2	Grade ≥3
*Major CVAEs*	*17 (14.7)*	*12*	*10*
ACS (STEMI)	1 (0.9)	0	1
ACS (NSTEMI)	3 (2.6)	0	3
Typical Chest pain	3 (2.6)	3	0
Heart failure	1 (0.9)	0	1
Dyspnoea post infusion	4 (3.5)	2	2
Syncope/pre-syncope	1 (0.9)	0	1
Arrhythmias	7 (6.0)	6	1
Sudden cardiac death	1 (0.9)	NA	1
*Hypertension-related CVAEs*	*45 (38.7)*	*75*	*29*
New onset/worsened hypertension	37 (31.9)	37	0
Masked hypertension	4 (3.5)	4	0
White coat hypertension	0 (0)	0	0
Pre-infusion uncontrolled hypertension [infusion limiting]	11 (9.5)	6	9
Pre-infusion uncontrolled hypertension [not-infusion limiting]	20 (17.2)	20	10
Post-infusion uncontrolled hypertension	11 (9.5)	8	6
Symptomatic uncontrolled hypertension	4 (3.5)	0	4
Hypertensive emergency	0 (0)	0	0
*All-type CVAEs*	*52 (44.9)*	*87*	*39*
*Both major and hypertensive CVAEs*	*10 (8.6)*	*26*	*13*

* patients experienced more than one CVAE, hence total % amount to > 100. CVAEs = cardiovascular adverse events; ACS = acute coronary syndrome; STEMI = ST-elevation myocardium infarction; NSTEMI = Non-ST-elevation myocardial infarction.

**Table 3:** Comparison between no-CVAEs and CVAEs groups: main baseline parameters.

Characteristics	No CVAEs No = 64 (53.4%)	CVAEs No = 52 (46.6%)	*p* Value
*Demographic and clinical*			
Age, median (SD), years	63.34 ± 8.26	66.00 ± 8.46	0.092
Male sex	36 (56.3)	29 (55.8)	0.959
*Individual CV risk factors, No (%)*			
Tobacco use (past/current)	29 (45.3)	29 (55.8)	0.263
Known Arterial Hypertension	25 (39.1)	23 (44.2)	0.574
Diabetes	7 (10.9)	5 (9.6)	0.816
Chronic renal failure (eGFR < 60 mL/m)	15 (23.4)	7 (13.5)	0.173
Coronary artery disease	1 (1.6)	3 (5.8)	0.217
Previous Atrial Fibrillation	2 (3.1)	0 (0)	0.501
Dyslipidaemia	10 (15.6)	6 (11.5)	0.526
Previous stroke	0 (0)	0 (0)	NA
Anti-hypertensive drugs ≥ 3	3 (4.7)	5 (9.6)	0.298
*Office BP values, mean (SD)*			
SBP, mmHg	124.71 ± 17.25	135.6 ± 16.87	**0.002**
DBP, mmHg	74.73 ± 12.39	79.35 ± 9.12	**0.024**
HR, bpm	77.22 ± 13.05	76.29 ± 13.49	0.720
*ABPM* *			
Daytime SBP, mean (SD), mmHg	122.98 ± 13.88	127.39 ± 12.15	**0.008**
Daytime DBP, mean (SD), mmHg	73.95 ± 9.40	76.37 ± 8.31	0.534
Daytime SD, mean (SD)	10.81 ± 3.80	15.14 ± 11.62	**0.009**
24 h SBP, mean (SD), mmHg	119.24 ± 13.48	122.94 ± 11.63	0.131
24 h DBP, mean (SD), mmHg	70.76 ± 8.38	72.73 ± 7.73	0.208
24 h MBP, mean (SD), mmHg	87.69 ± 9.54	89.39 ± 8.57	0.336
24 h SD, mean (SD)	12.43 ± 3.82	15.11± 4.64	**0.002**
Night time SD, mean (SD)	8.45 ± 2.82	10.22 ± 4.02	**0.011**
Dipping < 10%, No. (%)	25 (43.9)	13 (28.9)	0.120
Blood pressure variability, No. (%)	8.10 ± 2.47	10.31 ± 4.08	**0.001**
**Echocardiography** **^†^**			
LVMi, mean (SD), g/m^2^	85.30 ± 19.72	95.14 ± 21.75	**0.013**
LVEF, mean (SD), %*	63.03 ± 6.56	61.96 ± 7.13	0.414
GLS, mean (SD), %*	−22.37 ± 2.56	−21.3 ± 2.46	**0.029**
Diastolic dysfunction, No. (%)	1 (1.6)	0(0)	0.362
**Arterial Stiffness** **^‡^**			
PWV value, mean (SD), m/s	7.41 ± 1.63	8.55 ± 1.855	**0.002**

Mean values estimated in: * 107 patients; † 115 patients; ‡ 103 patients. *p* value < 0.05 highlighted in bold format. BMI = body mass index; eGFR = estimated glomerular filtration rate; ABPM = ambulatory blood pressure monitoring; SBP = systolic blood pressure; DBP = diastolic blood pressure; HR = heart rate; SD = standard deviation; MBP = mean blood pressure; LVMi = left ventricular mass indexed to body surface area; LVEF = left ventricular ejection fraction; GLS = global longitudinal strain; PWV = pulse wave velocity.

## Data Availability

Not applicable.

## Supplementary Materials

The following are available online at https://www.mdpi.com/article/10.3390/cancers13071631/s1, Table S1: Carfilzomib-based chemotherapeutic regimens, Table S2: Carfilzomib cumulative dose and time at the first all-type CVAE, major CVAE, hypertension-related CVAE and at the end of the planning therapy, Table S3: Comparison between no-CVAEs and CVAEs groups: additional baseline parameters.

## Appendix A

### Appendix A.1. Major Cardiovascular Adverse Events Definitions

Acute coronary syndromes, distinguished in ST and no-ST myocardium infarction, were diagnosed in presence of increase/decrease of a cardiac biomarker (with at least one value above the 99th percentile) and at least one of the following: symptoms, ischemic ECG changes, echocardiographic findings [32]. Heart failure was demonstrated by the presence of at least two of the three following items: symptoms and/or clinical signs, echocardiographic findings, elevated levels of natriuretic peptides [33]. Dyspnea related to CFZ-infusion was defined by a new onset of dyspnea within three days post CFZ-infusion, without clinical or instrumental signs of heart failure or pulmonary hypertension. Arrhythmias included atrial fibrillation or flutter, atrial bigeminy, ventricular tachycardia, ventricular bigeminy/trigeminy, and grade ≥ 2 of atrioventricular block. Typical chest pain was defined by the presence of chest pain with features suggestive of ischemic cardiac origin (duration, irradiation, onset, etc), and for this reason subjected to clinical and instrumental assessments (ECG, cardiac troponins and eventually echocardiography) with negative results. Syncope was defined by a spontaneous loss of consciousness, presyncope by dizziness and feeling faint (with or without hypotension) with evidence of cardiac origin at clinical and instrumental evaluations. Sudden cardiac death was defined by unexpected death during CFZ therapy without other proven causes.

### Appendix A.2. Arterial Hypertension Cardiovascular Adverse Events Definitions

New diagnosis of arterial hypertension was defined in presence of blood pressure values ≥140/90 mmHg at office or out-of-office BP measurements, in subjects prior normotensive (9); the worsening of known arterial hypertension was defined as increased BP values ≥140/90 mmHg requiring additional anti-hypertensive treatment in subject with known arterial hypertension. CFZ pre-infusion uncontrolled hypertension was defined by office BP values ≥140/90 mmHg within 30 minutes prior CFZ infusion, distinguished by limiting or permissive CFZ infusion. CFZ post-infusion uncontrolled hypertension was defined by office BP values ≥140/90 mmHg within 30 minutes from CFZ infusion. Masked hypertension was defined by the presence of arterial hypertension at ABPM when office BP measurements were normal; white coat hypertension was defined by normal ABPM values but elevated office BP [9]. Symptomatic uncontrolled blood pressure was demonstrated by symptomatic cases of BP >180/110 mmHg subjected to further assessment that excluded target acute organ damage; hypertensive emergency was defined by symptomatic BP >180/110 mmHg, with evidence of target acute organ damage at further assessment tests [9].
