# Supplementary material for: Multiple Myeloma Patients Undergoing Carfilzomib: Development and Validation of a Risk Score for Cardiovascular Adverse Events Prediction
Source: Cancers (Basel). 2021 Apr 1;13(7):1631. doi: 10.3390/cancers13071631 (PMC8036868; doi:10.3390/cancers13071631)
Supplement: Supplementary file 1 [file cancers-13-01631-s001.pdf]

cancers

MDPI

Article

Multiple Myeloma Patients Undergoing Carfilzomib: Development and Validation of a Risk Score for Cardiovascular Adverse Events Prediction

Anna Astarita [1,*], Giulia Mingrone [1], Lorenzo Airale [1], Fabrizio Vallelonga [1], Michele Covella [1], Cinzia Catarinella [1], Marco Cesareo [1], Giulia Bruno [1], Dario Leone [1], Carlo Giordana [1], Giusy Cetani [2], Marco Salvini [2], Francesca Gay [2], Sara Bringhen [2], Franco Rabbia [1], Franco Veglio [1] and Alberto Milan [1]

1 Hypertension Unit, Department of Medical Sciences, Division of Internal Medicine, AO "Città della Salute e della Scienza" University Hospital, Turin, 10126, Italy; giulia.mingrone@edu.unito.it (G.M.); lorenzo.airale@unito.it (L.A.); fabrizio.vallelonga@unito.it (F.Vallelonga); mcovella@ausl.vda.it (M.Covella); cinzia.catarinella@edu.unito.it (C.C.); marcoriccardo.cesareo@unito.it (M.Cesareo); giulia.bruno87@gmail.com (G.B.); dgleone@live.it (D.L.); carlo.giordana@edu.unito.it (C.G.); franco.rabbia@libero.it (F.R.); franco.veglio@unito.it (F.Veglio); alberto.milan@gmail.com (A.M.)

2 Myeloma Unit, Department of Medical Sciences, Division of Hematology, AO "Città della Salute e della Scienza" University Hospital, Turin, 10126, Italy; g.cetani@gmail.com (G.C.); marcosalvini83@gmail.com (M.S.); francesca.gay@unito.it (F.G.); sarabringhen@yahoo.com (S.B.)

* Correspondence: anna.astarita@unito.it. Tel.: 011-6336959

Citation: Astarita, A.; Mingrone, G.; Airale, L.; Vallelonga, F.; Covella, M.; Catarinella, C.; Cesareo, M.; Bruno, G.; Leone, D.; Giordana, C.; et al. Multiple Myeloma Patients Undergoing Carfilzomib: Development and Validation of a Risk Score for Cardiovascular Adverse Events Prediction. *Cancers* **2021**, *13*, 1631. https://doi.org/10.3390/cancers13071631

Academic Editors: Nicola Amodio

Received: date
Accepted: date
Published: date

Publisher's Note: MDPI stays neutral with regard to jurisdictional claims in published maps and institutional affiliations.

Copyright: © 2021 by the authors. Licensee MDPI, Basel, Switzerland. This article is an open access article distributed under the terms and conditions of the Creative Commons Attribution (CC BY) license (http://creativecommons.org/licenses/by/4.0/).

Supplementary Materials

Table S1. Carfilzomib-based chemotherapeutic regimens.

Protocol	Chemotherapeutic Agents	Administration days (Cycles of 28 days)	Patients No (%)*
KD	- Carfilzomib 56mg/m^2 (20mg/m^2 D1 and D2 of C1)<br>- Dexamethasone 20 mg	- D1, 2, 8, 9, 15, 16<br>- D1, 2, 8, 9, 15, 16, 22, 23	32 (27.5)
KRD	- Carfilzomib 27 mg/ m^2 (20mg/m^2 D1 and D2 of C1)<br>- Dexamethasone 40 mg<br>- Lenalidomide 25 mg	- D1, 2, 8, 9, 15, 16<br>(from C13: 1, 2, 15, 16)<br>- D1, 8, 15, 22<br>- From D1 to D21	53 (45.6)
FORTE<br>*Phase III study in newly diagnosed MM*	*Study arm 1 (ASCT after 4 cycles):*<br>- Carfilzomib 36 mg/m^2 (20mg D1 and D2 of C1)<br>- Dexamethasone 40 mg<br>- Lenalidomide 25 mg<br>*Study arm 2:*<br>- Carfilzomib 36 mg/m^2 (20mg D1 and D2 of C1)<br>- Dexamethasone 40 mg<br>- Lenalidomide 25 mg<br>*Study arm 3 (ASCT after 4 cycles):*<br>- Carfilzomib 36 mg/m^2 (20mg D1 and D2 of C1)<br>- Dexamethasone 20 mg<br>- Cyclophosphamide 300 mg/m^2	- D1, 2, 8, 9, 15, 16<br>- D1, 2, 8, 9, 15, 16, 22, 23<br>- From D1 to D21<br><br>- D1, 2, 8, 9, 15, 16<br>- D1, 2, 8, 9, 15, 16, 22, 23<br>- From D1 to D21<br><br>- D1, 2, 8, 9, 15, 16<br>- D1, 2, 8, 9, 15, 16, 22, 23<br>- D1, 8,15	6 (5.2)
EMN07<br>*Phase I/II study in RRMM*	- Carfilzomib 27/36/45/56 mg/m^2<br>(level -1/0/+1/+2) (20mg/m^2 D1 of C1)<br>- Dexamethasone 20 mg<br>- Pomalidomide 4 mg	- D1, 8, 15<br>- D1, 8, 15, 22<br>- From D1 to 21	13 (11.2)
ARROW<br>*RRMM*<br>*Phase III study in RRMM*	*Study arm A:*<br>- Carfilzomib 70 mg/m^2 (20mg/m^2 D1 of C1)<br>- Dexamethasone 40 mg<br>*Study arm B:*<br>- Carfilzomib 27 mg/m^2 (20mg/m^2 D1 and D2 of C1)<br>- Dexamethasone 40 mg	- D1, 8, 15<br>- D1, 8, 15 (22 from C1 to C9)<br><br>- D1, 2, 8, 9, 15, 16<br>- D1, 8, 15 (22 from C1 to C9)	3 (2.6)<br><br>3 (2.6)
EMN11<br>*Phase I/II study in RRMM*	(ASCT after 4 cycles)<br>- Carfilzomib 36 mg/m^2 (20mg/m^2 D1-D2 of C1)<br>- Dexamethasone 20 mg<br>- Pomalidomide 4 mg	- D1, 2, 8, 9, 15, 16<br>- D1, 2, 8, 9, 15, 16<br>- From D1 to D21	4 (3.5)

* 2 patients were treated with unspecified Carfilzomib-based regimen.

Table S2. Carfilzomib cumulative dose and time at the first all-type CVAE, major CVAE, hypertension-related CVAE and at the end of the planning therapy.

Event	Duration, median (SD), months	CFZ cumulative dose, median (SD), mg
First all-type CVAE	2.6 (0-49,6)	11125,0 (27,8 –7951,7) *
First Major CVAE	3.0 (0-21.2)	1730,9 (29,32- 5118,8) †
First Hypertension-related CVAE	3.7 (0-49.7)	860,0 (9,93-7951,7) ‡
End of CFZ regimen	8.6 (0.1-52.8)	2781,9 (29,4-1323.5) §

Data available for: *45 patients, †14 patients, ‡40 patients, §94 patients.

Table S3. Comparison between no-CVAEs and CVAEs groups: additional baseline parameters.

Characteristics	No CVAEs No=64 (53.4%)	No CVAEs No=52 (46.6%)	*p* value
Familiar CV risk factors			
Ischaemic cardiopathy, No (%)	17 (26.6)	9 (17.3)	0.235
Ictus cerebri, No. (%)	8 (12.5)	5 (9.6)	0.624
Diabetes, No. (%)	4 (6.3)	2 (3.8)	0.561
Arterial Hypertension, No. (%)	10 (15.6)	5 (9.6)	0.337
ECG			
Right Bundle Block, No. (%)	2 (3.6)	3 (7.7)	0.388
Left Bundle Block, No. (%)	1 (1.8)	0 (0)	0.397
Right and Left Bundle Block, No. (%)	2 (3.6)	2 (7.7)	0.388
LVH, No. (%)	6 (10.9)	6 (15.4)	0.522
Abnormal ST segment, No. (%)	6 (10.9)	6 (15.4)	0.522
Abnormal ECG, No. (%)	21 (38.9)	17 (42.5)	0.724
*Echocardiography**			
LVH, No. (%)	8 (12.7)	16 (30.8)	0.018
LV enlargement, No. (%)	5 (9.3)	4 (8.9)	0.949
Diastolic dysfunction, No. (%)	1 (1.6)	0(0)	0.362
LAVi enlargement, No. (%)	4 (6.3)	2 (3.8)	0.548
RV enlargement, No. (%)	6 (12.2)	5 (13.2)	0.899
TAPSE ≤ 17 mm, No. (%)	2 (3.5)	1 (2)	0.637
Previous Oncological Therapies, No. (%)			
Anthracyclines	16 (25.8)	15 (28.8)	0.716
Alkylating agents	53 (85.5)	38 (73.3)	0.100
Immunomodulating agents	46 (74.2)	40 (76.9)	0.736
Bortezomib	54 (87.1)	43 (82.7)	0.511
All	12 (19.4)	8 (15.4)	0.579

Mean values estimated on: *115 patients. LVH = left ventricular hypertrophy [computed according the Sokolow-Lyon Criteria: S wave in V1 plus the R wave in V5 or V6 > 35 mm]; LV enlargement = left ventricle enlargement [defined in case of LV diastolic diameter > 58 cm in males and > 52 cm in female]; LAVi = left atrial volume indexed to body surface area dilation [defined in case of LAVi volume ≥ 34 ml/m2]; RV dilation = right ventricle dilation [defined in case of RV basal diameter > 41 mm and/or RV mild diameter > 35 mm]; TAPSE = tricuspid annular plane excursion.
